# Cybersecurity Interventions in Health Care Organizations in Low- and Middle-Income Countries: Scoping Review

**DOI:** 10.2196/47311

**Published:** 2024-11-20

**Authors:** Kaede Hasegawa, Niki O'Brien, Mabel Prendergast, Chris Agape Ajah, Ana Luisa Neves, Saira Ghafur

**Affiliations:** 1 Institute of Global Health Innovation Imperial College London London United Kingdom; 2 Department of Primary Care and Public Health Imperial College London London United Kingdom; 3 RISE-Health Faculty of Medicine of the University of Porto Porto Portugal

**Keywords:** computer security, internet security, network security, digital health, digital health technology, cybersecurity, health data, global health, security, data science, LMIC, low income, low resource, scoping review, review methodology, implementation, barrier, facilitator

## Abstract

**Background:**

Health care organizations globally have seen a significant increase in the frequency of cyberattacks in recent years. Cyberattacks cause massive disruptions to health service delivery and directly impact patient safety through disruption and treatment delays. Given the increasing number of cyberattacks in low- and middle-income countries (LMICs), there is a need to explore the interventions put in place to plan for cyberattacks and develop cyber resilience.

**Objective:**

This study aimed to describe cybersecurity interventions, defined as any intervention to improve cybersecurity in a health care organization, including but not limited to organizational strategy(ies); policy(ies); protocol(s), incident plan(s), or assessment process(es); framework(s) or guidelines; and emergency planning, implemented in LMICs to date and to evaluate their impact on the likelihood and impact of attacks. The secondary objective was to describe the main barriers and facilitators for the implementation of such interventions, where reported.

**Methods:**

A systematic search of the literature published between January 2017 and July 2024 was performed on Ovid Medline, Embase, Global Health, and Scopus using a combination of controlled terms and free text. A search of the gray literature within the same time parameters was undertaken on the websites of relevant stakeholder organizations to identify possible additional studies that met the inclusion criteria. Findings from included papers were mapped against the dimensions of the Essentials of Cybersecurity in Health Care Organizations (ECHO) framework and presented as a narrative synthesis.

**Results:**

We included 20 studies in this review. The sample size of the majority of studies (13/20, 65%) was 1 facility to 5 facilities, and the studies were conducted in 14 countries. Studies were categorized into the thematic dimensions of the ECHO framework, including context; governance; organizational strategy; risk management; awareness, education, and training; and technical capabilities. Few studies (6/20, 30%) discussed cybersecurity intervention(s) as the primary focus of the paper; therefore, information on intervention(s) implemented had to be deduced. There was no attempt to report on the impact and outcomes in all papers except one. Facilitators and barriers identified were grouped and presented across national or regional, organizational, and individual staff levels.

**Conclusions:**

This scoping review’s findings highlight the limited body of research published on cybersecurity interventions implemented in health care organizations in LMICs and large heterogeneity across existing studies in interventions, research objectives, methods, and outcome measures used. Although complex and challenging, future research should specifically focus on the evaluation of cybersecurity interventions and their impact in order to build a robust evidence base to inform evidence-based policy and practice.

## Introduction

### Background

Health systems globally are incorporating technology into every aspect of the delivery of care [1]. The health sector is also one of the most targeted and profitable sectors for cyberattacks [2], defined as the malicious attempt to gain unauthorized access to online systems or computers [3]. The United States National Institute of Standards and Technology (NIST) expands upon this definition, describing a cyberattack as “any kind of malicious activity that attempts to collect, disrupt, deny, degrade, or destroy information system resources or the information itself” [4]. Medical records are more valuable than credit card details on the dark web in some countries, due to the inclusion of both patient identifiers and financial information [5]. Health care organizations globally have seen a significant increase in the frequency of cyberattacks in recent years as technology plays more of a central role in the delivery of care [6,7].

In 2017, the WannaCry malware attack targeted computers running with an unsupported Microsoft Windows operating system [8]. The impact on the UK health system was far-reaching, and the incident remains the largest cyberattack to affect the National Health Service (NHS), with 34 hospital trusts and 603 primary care and other NHS organizations directly affected [8]. In 2021, Ireland’s health service faced a ransomware attack after an employee in the Irish Health Service Executive opened a spreadsheet email attachment compromised with malware [9]. In 2023, Pennsylvania Lehigh Valley Health Network was also the target of a ransomware attack in which the clinical images of individuals receiving cancer treatment were released [10]. These attacks serve only as examples of the many cyberattacks the health sector has faced. Health care cybersecurity is underpinned by staff understanding of the significance of threats, as most security breaches occur through human error.

Cyberattacks not only cause massive disruption to health service delivery but also directly impact patient safety. They disrupt and delay treatment, as well as threaten the safe use of personal data, which can lead to mistrust by patients and the public toward health care providers [11]. In extreme cases, cyberattacks may lead to patient morbidity and mortality. For example, analysis of the impacts of the UK NHS WannaCry attack showed a 6% decrease in total admissions per infected hospital per day during WannaCry, with 4% fewer emergency admissions and 9% fewer elective admissions [12]. Research has shown that delays to hospital inpatient admissions in excess of 5 hours from time of arrival at the emergency department are associated with an increase in all-cause 30-day mortality. Furthermore, for every 82 admitted patients whose time to inpatient bed transfer is delayed beyond 6 hours to 8 hours from arrival time, there is 1 additional death [13]. In a separate study, delayed elective surgery patients also reported a loss of working days for themselves and family members; increased disappointment, frustration, and stress; concern for continued symptoms; and deteriorating conditions [14]. Such downstream impacts outline the potential far-reaching impacts cybersecurity attacks and the disruption caused have on patients.

Health care cybersecurity is increasingly relevant to health systems globally, including in low- and middle-income countries (LMICs). In LMICs, the implementation of digital technologies in health care service provision is rapidly accelerating [15] and becoming an increasing part of the health agenda [7]. Health care systems in LMICs are progressively using digital innovations, including health care informatics systems, electronic health care records, and wearables [11].

Health systems in LMICs have also seen an increase in cyber threats [3]. East Asia Saraburi Hospital in Thailand was the target of a ransomware attack in 2020, followed by another cyberattack on the Public Health Ministry. Together, these resulted in the potential theft of 16 million patient records [16]. The incidents led the Thai National Cyber Security Agency to announce 3 subordinate laws under the Cybersecurity Act to develop stronger cybersecurity. Among these laws was a mandate to provide cybersecurity training and conduct risk assessments for those dealing with critical information [16]. Similarly, Life Healthcare, a private provider in South Africa, was attacked in 2020, disrupting the admissions systems, business processing systems, and email servers, though patient care was not impacted due to a swift switch to backup systems [17].

Despite the increase in cyberattacks in the health sector in LMICs, there is limited evidence on the number and nature of cyberattacks as well as interventions put in place to plan for cyberattacks and develop cyber resilience in these settings. Cybersecurity interventions are defined as any intervention to improve cybersecurity in a health care organization. These include but are not limited to organizational strategy(ies); policy(ies) (eg, policy for installation of appropriate malware and virus protection software, access management policy); protocol(s), incident plan(s), or process(es) (eg, processes around identity management, threat detection); framework(s) or guidelines; education (eg, courses, training); and emergency planning. Given increasing cybersecurity threats, it is essential to develop a knowledge base on interventions found to be effective at improving cyber preparedness in the health sector. A first step in achieving this is to understand which interventions have been developed and what their impact has been,

### Objectives

The primary objectives of this scoping review were to (1) describe cybersecurity interventions implemented in LMICs to date and (2) evaluate their impact on the likelihood and impact of attacks. As a secondary objective, we aimed to identify the main barriers and facilitators for the implementation of such interventions, where reported.

## Methods

This scoping review followed the PRISMA (Preferred Reporting Items for Systematic Reviews and Meta-Analyses) reporting guidelines [18].

### Search Strategy

A systematic search of the literature published between January 2017 and July 2024 (current) was performed on Ovid Medline, Embase, Global Health, and Scopus using a combination of controlled terms and free text, depending on the database functionality. The detailed search strategy is provided in Multimedia Appendix 1. Searches were conducted in 3 health-specific (Ovid Medline, Embase, and Global Health) databases, and as such, no health care keywords were used in the search. Given the multidisciplinary topic area and the importance of capturing literature from information and communication technology (ICT) and computer science disciplines, Scopus was also searched with specific health care keywords (“health,” “healthcare,” “medical,” “hospital,” and “clinic”). We selected Scopus because the IEEE Xplore Library, a leading source of cybersecurity-focused research, is indexed by the database.

A search of the gray literature within the same time parameters was undertaken to identify possible additional studies that met the inclusion criteria. Searches were conducted on the websites of relevant stakeholder organizations (ie, the World Bank and the World Health Organization [WHO] including WHO regional offices and the WHO International Clinical Trials Registry Platform Search Portal), Ponemon Institute, Cybersecurity and Infrastructure Security Agency, CyberPeace Institute, Gartner, and Verizon) and conference proceedings from related conferences (ie, Healthcare Information and Management Systems Society, Global Forum on Cyber Expertise).

### Selection Criteria

We included any health care–focused study undertaken in (1) an LMIC country that (2) described 1 or multiple interventions related to cyberattacks or cybersecurity (Textbox 1). Only studies published in English were included. Feasibility studies, commentaries, and editorial papers were excluded.

**Inclusion and exclusion criteria.**

Article type

- Inclusion criterion: original research study, including systematic reviews
- Exclusion criteria: feasibility studies, commentaries, and editorial papers

Language

- Inclusion criterion: English
- Exclusion criterion: any other language

LMICs are defined by the World Bank based on gross national income per capita [19]; however, it has been noted that the categorization should only be used when relevant to the research study [20]. Health systems in LMICs are often challenged by resourcing constraints at a national level, which filter down to local systems and providers, and, as such, likely face common resourcing challenges for developing and implementing cybersecurity interventions. Given these shared challenges, focusing the research on LMICs as a general group was agreed by the authors. Notably, our use of the term serves as a starting point to explore cybersecurity interventions rather than an end point, as further nuanced research and evidence generation will be required.

Cybersecurity interventions were defined as any intervention to improve cybersecurity in a health care organization, including but not limited to organizational strategy(ies); policy(ies); protocol(s), incident plan(s), or assessment process(es); framework(s) or guidelines; education (eg, courses, training); and emergency planning.

Title and abstract screening followed by full-text screening were performed by 3 independent researchers (NO, KH, CAA) based on the inclusion and exclusion criteria described. Conflicts were resolved by consensus at each stage of the screening process. Intercoder agreement was measured by calculating the Cohen kappa at each screening phase. The quality of included publications was not assessed.

### Data Extraction and Analysis

Studies that met the inclusion criteria were extracted by the first independent researcher (KH) using a standardized Microsoft Excel spreadsheet and reviewed by 2 other independent researchers (NO, ALN) to ensure data quality and consistency. Data extracted included author names, year of publication, study design, setting, population, outcome measures, and main findings.

### Analysis Framework

Identified cybersecurity interventions were mapped against the Essentials of Cybersecurity in Health Care Organizations (ECHO) framework, a guide for policymakers and health and care organizations to strengthen their cybersecurity infrastructure, which positions cybersecurity interventions across 6 dimensions including context, the wider conditions within which organizations’ cyber planning operates; governance, which includes the policies and protocols to reduce the threat of cyberattacks; organizational strategy, involving policies, planning, and the allocation of responsibility at the organizational level; risk management, which involves identifying, assessing, and mitigating cyber risks; awareness, education, and training, which are actions to ensure that all stakeholders within the organization have at least core knowledge on cybersecurity; and technical capabilities, or technical requirements needed to safeguard cybersecurity [21,22]. The ECHO framework was developed in 2020 as a health sector–specific framework for ICT professionals and nonexpert stakeholders. The was recently included as a recommended resource in the World Bank’s knowledge notes series, entitled “Implementation Know-how Briefs to Support Countries to Prioritize, Connect and Scale for a Digital-in-Health Future (Cybersecurity in health brief)” [23].

A narrative synthesis of the findings was conducted. Relevant findings and outcome measures were grouped into subcategories and organized based on the 6 dimensions of the ECHO framework. Due to heterogeneity of the populations, interventions, comparators, and outcomes across the included studies, a meta-analysis was not performed, and no attempt was made to compare cybersecurity interventions described.

## Results

### Studies Included in the Analysis

**Figure 1:**
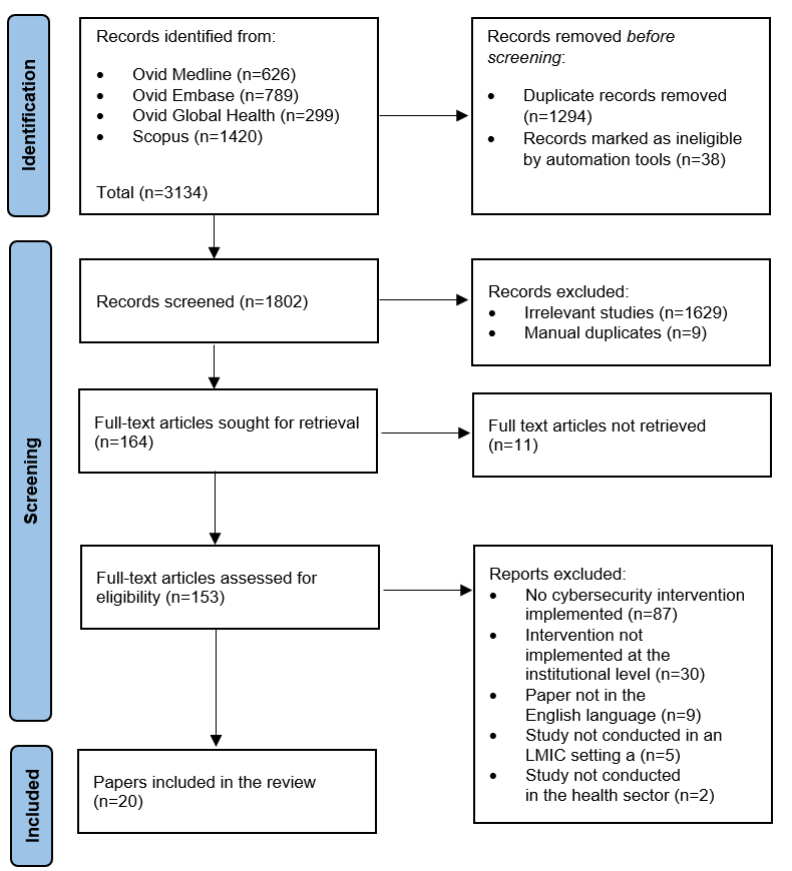
PRISMA (Preferred Reporting Items for Systematic Reviews and Meta-Analyses) flowchart. LMIC: low- and middle-income country.

The database search identified a total of 3134 publications. After removal of 1294 duplicates and 38 publications marked as ineligible by automation tools (eg, Covidence automatic deduplication function), 1802 papers underwent screening. Of these, 164 studies underwent full-text screening, and 20 eventually were finally included in the review (Figure 1). Cohen kappa values were 0.29 in the abstract screening and 0.64 in the full-text review.

### Description of Included Studies

Table 1 presents the country and economic classification status of each of the papers. Notably 1 research study was conducted in both Libya and Yemen, so this was counted as 2 studies for this analysis, resulting in 21 total studies. The countries most represented in the studies included were Peru (3/21, 14%) and South Africa (3/21, 14%), followed by China, Iran, and Indonesia (2/21, 10% each). All other countries included (Brazil, Ecuador, Ghana, Indonesia, Jordan, Libya, Nepal, Turkey, Vietnam, Yemen) were represented in only 1 study each (1/21, 5%). Two-thirds (14/21, 67%) of the studies were conducted in upper middle-income countries, and 6 (6/21, 29%) were conducted in lower-middle income countries, with 1 (5%) conducted in a low-income country setting. One study was conducted in 2 countries (Libya and Yemen). The sample size (ie, number of institutions involved in the study) ranged from 1 facility to 312 facilities: 7 (7/20, 35%) studies were based in a single facility, 6 studies (6/20, 30%) were conducted in 2 facilities to 5 facilities, and 7 (7/20, 35%) studies were conducted in 6 or more facilities.

**Table 1:** Description of studies by country, World Bank economic classification, and number of facilities.

Characteristic		Studies, n (%)
**Country**		
	Peru	3 (14)
	South Africa	3 (14)
	China	2 (10)
	Indonesia	2 (10)
	Iran	2 (10)
	Brazil	1 (5)
	Ecuador	1 (5)
	Ghana	1 (5)
	Jordan	1 (5)
	Libya	1 (5)
	Nepal	1 (5)
	Turkey	1 (5)
	Vietnam	1 (5)
	Yemen	1 (5)
**Economic classification^a^**		
	UMIC^b^	14 (67)
	LMIC^c^	6 (29)
	LIC^d^	1 (5)
**Number of facilities**		
	1	7 (35)
	2-5	6 (30)
	≥6	7 (35)

**^a^**Country status based on the World Bank economic classification [19].

^b^UMIC: upper middle-income country.

^c^LMIC: low- and middle-income country.

^d^LIC: low-income country.

### Description of Cybersecurity Interventions Implemented in LMICs

Of the 20 studies, only 6 (30%) discussed the intervention(s) as the primary focus of the paper. Information on intervention(s) implemented had to be deduced from the remaining papers through analysis of responses to staff or hospital-level surveys (12/20, 60%) and case studies (1/20, 5%). The gray literature search undertaken with the same parameters did not find any additional papers to include. Detailed information of the included studies, including a description of cybersecurity intervention(s), is presented in Table 2. Technical capabilities (14/55, 25%) and awareness, education, and training (11/55, 20%) were the 2 most common interventions mentioned in the studies, followed by governance (9/55, 16%), organizational strategy (9/55, 16%), risk management (7/55, 13%), and context (5/55, 9%). Table 3 presents the description of studies by ECHO framework dimension.

**Table 2:** Detailed information (authors, year, country, institution, intervention) of the included studies.

Authors	Year	Country(s)	Institution name	Description of intervention
Ali Alferjanya et al [24]	2022	Libya, Yemen	Not stated; 5 private hospitals in Libya and the 5 biggest private hospitals in Yemen	No direct discussion of intervention; some of the organizational staff survey reported attendance of ICT^a^ security training. Similarly, some staff reported the use of “different passwords across multiple web portals, systems, or applications” suggesting organizational technology-based cybersecurity measures, such as access control.
Ayatollahi and Shagerdi [25]	2017	Iran	Not stated; 27 hospitals located in 1 city in northwest Iran	Organizational staff reported on information security safeguards, including technical, administrative, and physical safeguards. The most common technical safeguards included access control and user authentication and detective control tests.
Chuma and Ngoepe [26]	2022	South Africa	Not stated; 1 public hospital in South Africa	Direct discussion of interventions; an organizational staff survey found the adoption of relevant legislation and the ISO27799 standard. The hospital established policies such as an administrative policy, records management policy, and medicine policy but had no formal security policy. Technical safeguards implemented at the hospital included password and username, data encryption, firewall and antivirus, and risk management activities such as maintaining a security audit log.
Daraghmeh and Brown [27]	2021	Jordan	Not stated; 39 private hospitals in Jordan	Organizational staff reported on various cybersecurity interventions. Some hospitals reported complying with relevant regulations and standards; having business continuity, disaster recovery, and risk management plans in place; and measuring the maturity of cyber measures. Some hospitals also reported requiring their staff to undergo training and various technological measures, including patching, firewalls, and authentication.
Dönmez et al [28]	2020	Turkey	Not stated; 15 public health institutions in Kocaeli, Marmara region	No direct discussion of intervention; organizational staff survey found attendance at ICT security training. Similarly, some staff reported “access to the information security policy documents at the hospital,” suggesting organizational planning and strategy; “Personal information is stored via information technologies securely”; and password management systems, suggesting technical controls in some institutions.
Hou et al [29]	2018	China	Not stated; 1 public hospital in Shaanxi province, China	No direct discussion of intervention; an in-depth case study presented the hospital's information security management measures, including regulatory and standards adherence, risk management activities and procedures, staff information security awareness training, technical controls, and organizational security strategies.
Jara et al [30]	2021	Peru	Not stated; 1 private hospital in Lima, Peru	Implementation of a model of cybersecurity capability measurement, calculated by the level of compliance with controls
Kandabongee Yeng et al [31]	2023	Ghana	Not stated; 2 hospitals in Ghana	No direct discussion of intervention; staff “shared their knowledge of security measures related to password use, access control, vulnerability reporting and logging of users’ access” as part of the research methodology, suggesting these interventions were in place at the 2 hospitals. Additionally, ICT and administration staff shared their knowledge on security governance, virus control, data backup, and training. Hospital A also reported having a draft security policy.
Khac Hai et al [32]	2017	Vietnam	Not stated; 312 HIV outpatient clinics in Vietnam	No direct discussion of intervention; some of the organizational staff survey reported attendance at ICT security training.
Maeko and Van Der Haar [33]	2018	South Africa	Charlotte Maxeke Academic Hospital, Johannesburg, South Africa	No direct discussion of intervention; some staff reported adhering “to the control procedures of locking or logging off their computers,” suggesting organizational technology-based cybersecurity measures, such as access control, and some reported attendance at ICT security training.
Meng et al [34]	2018	China	1 anonymous organization in China implemented the approach	Implementation of a trust-based approach based on Bayesian inference to identify malicious devices in a health care environment
Nistrina and Bin Bon [35]	2019	Indonesia	Soreang Hospital, Bandung, Indonesia	Undertaking of a maturity assessment based on the COBIT^b^ 5 capability levels
Quimiz-Moreira et al [36]	2022	Ecuador	Verdi Cevallos Balda Hospital, IESS^c^ Hospital, and Rodríguez Zambrano Hospital, Ecuador	No direct discussion of intervention; some of the hospitals reported on the use of 14 cybersecurity intervention areas including information security policies, roles and responsibilities, organization of information security, safety linked to human resources, asset management, access control, management of patient data (eg, pseudonymization), physical security of ICT equipment, operations security (eg, back-up, event logging), communications security (eg, network security management), acquisition, development and maintenance of information systems, supplier and third-party policies, incident management planning, business continuity, and compliance with security standards, policies, laws, and regulations.
Rijo et al [37]	2020	Brazil	Not stated; 11 hospitals in Brazil	No direct discussion of intervention; some of the hospitals surveyed reported on the use of 12 aspects of cybersecurity: IT security (eg, installing security patches), interoperability, hardware infrastructure, network infrastructure, business process management, use of standards, use of ISO^d^ standards and certifications, research and development activities, IT team profile, alignment with strategy, decision-making and procurement, IT investment.
Serrano Rojas et al [38]	2022	Peru	Not stated; 1 health center in Lima, Peru	Implementation of a cybersecurity maturity model for health data privacy and protection
Sanchez Rubio et al [39]	2022	Peru	Not stated; 2 clinics in Lima, Peru	Implementation of a model to identify the level of maturity of the health clinics for preventing data leak scenarios
Sarbaz et al [40]	2019	Iran	Not stated; 12 academic hospitals in Mashhad, northeastern Iran	No direct discussion of intervention; some of the organizational staff survey reported the provision of information security training for staff and use of access control methods within their hospitals. Similarly, some staff reported “My organization runs security controls (such as firewall, antivirus, encryption, etc.) to protect sensitive information” and “My organization has used security procedures to protect important information from being stolen by malware (such as decoder, trojans, and spyware),” suggesting organizational technology-based cybersecurity measures and organizational planning.
Kencana Sari et al [41]	2023	Indonesia	National Cardiovascular Center Harapan Kita (RSPJNHK), a specialized public hospital	No direct discussion of intervention; implementation of interventions was mapped against ISO 27799:2016, and staff surveys were undertaken. The listed interventions included HIS^e^ implementation policy, including information security management, set up of a System Maintenance and Security Sub-unit in the IT Department, establishment of procedures for incident reporting, ICT security training, access controls, establishment of a safe area for the data center room, and various technological measures, including anti-virus, firewalls, and authentication.
Singh and Singh [42]	2022	South Africa	Not stated; 2 hospitals in northern Kwa-Zulu Natal, South Africa	No direct discussion of intervention; some of the organizational staff survey reported attendance at ICT security training. Most reported this training taking place at the hospital, though others reported training at school or a university or college.
Upadhyaya et al [43]	2018	Nepal	Tertiary hospital for Children, Eye, ENT^f^ and Rehabilitation Services and associated community hospital in Chapagaun, Nepal	Implementation of a centralized, permissioned blockchain-based, secured health care data system using hyperledger alongside information security measures including training and adoption of quality and safety standards

^a^ICT: information and communication technology.

^b^COBIT: Control Objectives for Information and Related Technologies.

^c^IESS: Hospital de Especialidades Carlos Andrade Marín.

^d^ISO: International Organization for Standardization.

^e^HIS: health information system.

^f^ENT: ear, nose, throat.

**Table 3:** Mapping of the 20 included studies against the dimensions of the Essentials of Cybersecurity in Health Care Organizations (ECHO) framework.

ECHO dimension	Studies including the dimension, n (%)^a^	Papers
D1: Context	5 (9)	Daraghmeh and Brown [27], Jara et al [30], Nistrina and Bin Bon [35], Serrano Rojas et al [38], Sanchez Rubio et al [39]
D2: Governance	9 (16)	Chuma and Ngoepe [26], Daraghmeh and Brown [27], Hou et al [29], Kandabongee Yeng et al [31], Quimiz-Moreira et al [36], Rijo et al [37], Sarbaz et al [40], Kencana Sari et al [41], Upadhyaya et al [43]
D3: Organizational strategy	9 (16)	Chuma and Ngoepe [26], Daraghmeh and Brown [27], Dönmez et al [28], Hou et al [29], Kandabongee Yeng et al [31], Quimiz-Moreira et al [36], Rijo et al [37], Sarbaz et al [40], Kencana Sari et al [41]
D4: Risk management	7 (13)	Ayatollahi and Shagerdi [25], Chuma and Ngoepe [26], Daraghmeh and Brown [27], Hou et al [29], Quimiz-Moreira et al [36], Rijo et al [37], Sarbaz et al [40]
D5: Awareness, education, and training	11 (20)	Ali Alferjanya et al [24], Daraghmeh and Brown [27], Hou et al [29], Kandabongee Yeng et al [31], Khac Hai et al [32], Maeko and Van Der Haar [33], Quimiz-Moreira et al [36], Sarbaz et al [40], Kencana Sari et al [41], Singh and Singh [42], Upadhyaya et al [43]
D6: Technical capabilities	14 (25)	Ali Alferjanya et al [24], Ayatollahi and Shagerdi [25], Chuma and Ngoepe [26], Daraghmeh and Brown [27], Dönmez et al [28], Hou et al [29], Kandabongee Yeng et al [31], Maeko and Van Der Haar [33], Meng et al [34], Quimiz-Moreira et al [36], Rijo et al [37], Sarbaz et al [40], Kencana Sari et al [41], Upadhyaya et al [43]

^a^Some studies mentioned multiple interventions that corresponded to different dimensions of the ECHO framework, resulting in an N of 55.

### Impact on Interventions of the Likelihood and Impact of Attacks, Clinical Outcomes, or the Quality and Safety of Care

Of the 6 studies that described the implementation of interventions, 5 did not report on outcomes, either related to impact on frequency or scale of cyberattacks, clinical outcomes, or the quality and safety of care; therefore, the impact remains unknown [26,30,34,35,38]. One study, however [43], reported on the impact of a blockchain-based, secured health care data system using hyperledger alongside information security measures (the intervention) on patient satisfaction and noted that the health care organization “did not undergo repetitive work like history taking, investigations etc.,” suggesting that administrative waste was decreased, a key component of efficiency as described by the Institute of Medicine Six Domains of Health Quality Care [44]. There was no attempt to report on the impact and outcomes following the interventions in the remaining 14 papers from which interventions had to be deduced.

### Main Barriers and Facilitators for the Implementation of Cybersecurity Interventions

The main facilitators and barriers identified are presented in Table 4. Facilitators to implementation were directly reported in only 1 study [43]. Upadhyaya et al [43] noted success factors (facilitators) in the implementation of a blockchain-based, secured health care data system, which included ease of use, improved security and reliability, and training to develop knowledge on its use to enhance diagnosis and treatment.

**Table 4:** Main facilitators and barriers to implementation identified from the 20 included studies.

Level	Facilitators	Barriers
National or regional	Clear national legislation and policies (supported in organizations by active monitoring, evaluation, and learning)	Lack of national or regional policies and guidance for organizations Threat of natural disasters
Organizational	Staff engagement and training Management support	Lack of knowledge among staff Lack of ICT^a^ experts Exclusion of top-level managers Cost of implementation Political or high-level influence
Individual	Perception of the importance of cybersecurity Intervention’s ease of use Security and reliability of intervention	Disregard of cultural differences Lack of time

^a^ICT: information and communication technology.

Facilitators to implementation of cybersecurity interventions could be deduced from the discussion in 11 studies but were not directly reported [24,26,28-34,41,42]. At an organizational level, one of the main facilitators found in 4 studies was that national legislation and policies enabled health care organizations to create their own information security management guidelines and subsequent interventions [26,29,33,34]. However, national legislation and policy was most effective when it was supported by active monitoring and penalization, for example with hospital inspections [26,29]. Jara et al [30] suggested that routine collection of data on information security by organizations enabled better cybersecurity practices due to a continuous process of evaluation and improvement. In addition, the most important factors for positive staff engagement with cybersecurity software are reportedly the appearance features, screen interface, and volume of information on the screen [42]. At an individual level, one of the main facilitators identified by 4 studies was that a perception of the importance of cybersecurity positively influenced cybersecurity intervention adherence [28,31-33]. This perception was influenced by different factors; for instance, Dönmez et al [28] suggested that health care workers recognized the need for improved cybersecurity, while Maeko and Van Der Haar [33] suggested that health care workers recognized the importance of safeguarding their patients. Studies recognized that a facilitator for successful cybersecurity training was when employees created their own cybersecurity goals and action plans [24,29]. Ali Alferjanya et al [24] expanded on this point by suggesting that this was facilitated by the inclusion of staff supervisors to aid staff with creating their goals.

Barriers to implementation were directly reported in 2 studies [25,41]. Maeko and Van Der Haar [33] noted barriers associated with awareness, education, and training and technical capability–focused interventions. Cost was perceived by 49% of respondents as a major barrier to training being offered at the hospital, and implementation barriers associated with multimodal access control systems included cost; political influence, as high-level decision-making can result in employee resistance; and existing cyber hygiene practices. Kencana Sari et al [41] noted that barriers associated with health care worker adoption of security behaviors included perceived downsides (eg, multifactor authentication taking more time) and workload constraints. Barriers to implementation of cybersecurity interventions could be deduced from the discussion in 9 studies [24-26,29,32-34,40,42]. One of the main barriers to implementing interventions was the lack of knowledge among all levels of health care staff [24,32-34,40,42]. Hou et al [29] noted that a barrier to effective training is that national policies do not outline how information security awareness training should be carried out. Meng et al [34] also mentioned that a lack of IT experts within health care organizations hindered cybersecurity practice. With regards to the successful implementation of cybersecurity training, disregard for cultural differences, a lack of time among health care staff, and exclusion of top-level managers were identified as major barriers [24,29,33]. The threat of natural and man-made disasters such as floods and fires, specifically electrical fires that impact electric power transmission, were found to be additional barriers [25,26]. Finally, Chuma and Ngoepe [26] identified a lack of resources such as power and system failures, poor network, and outdated systems as key challenges.

## Discussion

### Main Findings

The primary outcome of this scoping review was the description of cybersecurity interventions implemented in the LMIC health care organizations described in the 20 studies. Our results indicate that there is limited research focusing directly on identifying and evaluating cybersecurity interventions in our research context. Only 6 of the 20 studies focused directly on cybersecurity interventions [26,30,34,35,38,43]. The remaining 14 studies included an indirect discussion by using surveys or questionnaires to investigate the state of cybersecurity in health care organizations [24,25,27-29,31-33,36,37,39-42].

From the included studies, which directly investigated cybersecurity interventions [26,30,34,35,38,43], 1 study proposed a way of calculating devices’ trust values and identifying trusted devices by means of a Bayesian inference approach, and another implemented a blockchain-based, secured health care system to transfer data. Five studies focused on ECHO Dimension 1, specifically investigating cybersecurity capability and maturity, implementing models and frameworks to assess security capabilities [27,30,35,38,39], with 1 study using an existing framework called Control Objectives for Information and Related Technologies (COBIT) 5 [35].The studies that indirectly discussed cybersecurity interventions through surveys or questionnaires most frequently described cybersecurity staff training (ECHO Dimension 5) as an intervention, followed by access control, including use of a password and user authentication (ECHO Dimension 6). Only 1 study reported on impact and outcomes of the interventions, specifically on patient satisfaction.

Despite the high frequency of staff training interventions being described, one of the main barriers mentioned was a lack of cybersecurity knowledge among health care staff [24,32-34,40,42]. This may indicate that existing ICT training is either sporadic or not effective in educating its staff members on cybersecurity and their roles and responsibilities. In this context, it is important to note that evidence is contradictory in what concerns the effectiveness of ICT training [44-46]. One of the main facilitators of training engagement was when staff were aware of the value of training and its importance to their patients [28,32,33]. This could suggest that the problem lies within the health care culture, rather than the training itself. We recommend that interventions should hold a larger focus on changing the culture around the importance of cybersecurity. Given that cybersecurity training is a nontechnical intervention that most staff will be exposed to, it is also likely that this was overreported in staff surveys.

National and regional policy and legislation play an important role in creating a setting for cybersecurity interventions to be implemented [26,29,33,34]. The facilitators of cybersecurity interventions mentioned in the studies often suggested that national legislation on cybersecurity gives health care organizations a direction for the development and implementation of their own policies and interventions. Hou et al [29] specifically mentioned that one of the barriers to effective cybersecurity staff training is that national policy does not outline how information security awareness training should be carried out. It is recommended that health care organizations in LMICs collaborate with government stakeholders not only in the development of national and regional policy but also in the implementation of cybersecurity interventions. For instance, in the United Kingdom, the NHS England cybersecurity team collaborates with the UK National Cyber Security Centre on interventions to outline what effective digital practice is [47]. LMICs can similarly build on their national policies and collaborate with governments to develop effective cybersecurity interventions.

There was a spread of World Bank economic classifications in the country settings across the included studies. However, upper middle-income countries were most represented, as it is likely that they generally have greater access to resources and technology, as compared with LMIC contexts. Furthermore, 5 of the 20 studies were conducted in private hospitals [24,27,30,37,39], with Daraghmeh and Brown [27] performing a study of 39 private hospitals. These settings are not fully representative of the low-resource contexts in which many cybersecurity interventions must be implemented in public, non-for-profit, and faith-based facilities. More studies need to be conducted in low-resource settings that directly measure the impact of cybersecurity interventions.

### Comparison With the Previous Literature

Findings of this scoping review are consistent with previous evidence suggesting that health care organizations globally are scaling up their responses to cyberattacks, particularly in the post-COVID-19 pandemic context [48,49]. However, it is notable that the limited evidence focused on investigation within LMIC health systems makes an in-depth comparison with previous literature in LMICs challenging. In the global context, including high-income country (HIC) health systems, He et al [48] also found that interventions used in the health sector during COVID-19 included increasing security awareness, enabling business continuity, and applying technical controls.

As Table 4 indicates, there were a range of facilitators and barriers to the implementation of cybersecurity interventions in LMIC settings reported or deduced from the evidence base. These findings are consistent with research undertaken in HICs. Coventry et al [50] found that barriers to secure behavior in health care among staff at 3 health care sites in Ireland, Italy, and Greece were a result of a lack of policies and reinforcement of secure behavior, poor awareness of consequences, and security as a barrier to productivity or patient care. Branley-Bell et al [51] also noted time pressures and fatigue as barriers to secure behavior in the health care context. Financial barriers to implementation of cybersecurity interventions identified in this study were also echoed in findings from interviews with cybersecurity experts working in hospitals in Canada and the United States [52]. Notably, a lack of comparable research on facilitators suggests more research is required across HIC and LMIC contexts.

Given the nascent literature on the impact of interventions on likelihood and impact of attacks, clinical outcomes, or the quality and safety of care in the wider literature, analysis of the 1 study outlining an impact on patient satisfaction and efficiency [43] remains limited. It is hoped that the evidence gap will be addressed given the increasing incidences of cyberattacks in health care and the growing use of digital technologies in health service delivery, making cybersecurity an essential element of patient safety in the health sector context.

### Strengths and Limitations

This scoping review has several strengths [53]. The review represents the first known attempt to gather information systematically on cybersecurity interventions in health care organizations in LMICs. The broad nature of the scoping review methodology enabled the inclusion of a range of studies with varied aims and using varied methods. The search terms also contributed to the inclusion of 17 papers, enabled discussion on the topic of interest, and identified facilitators and barriers to the implementation of cybersecurity initiatives.

This review has several limitations. First, the scoping review was not registered in a database (eg, PROSPERO) because few results were expected to have detailed information to enable the application of synthesis methods to determine outcomes and effects. It is possible that some papers were missed in the search. However, we sought to mitigate this limitation by developing broad search terms, screening multiple databases both inside and outside medicine and health care, involving a team in the screening process, checking references in the included papers, and undertaking a search of the gray literature. Although care was taken to ensure that technical cybersecurity publications were captured by searching Scopus, some papers may have been missed as the Scopus catalog provides only 89% coverage of the ACM Digital Library, a common database of cybersecurity results. There was no assessment of bias in the included studies. Given our inclusion and exclusion criteria, it is likely that non-English studies published on this topic were missed. This limitation is particularly pertinent as a large number of LMICs are non-English speaking.

### Implications for Research and Policy

This scoping review is the first of its kind and identifies a critical lack of investigation on cybersecurity interventions implemented in health care organizations. One of the main gaps in the literature is the direct evaluation of interventions, including the assessment of outcomes and impact, and studies focusing primarily on this as their main focus. As such, there is a need for robust evaluation of impact and outcomes. It is also important that further research is conducted across public, private, not-for-profit, and faith-based health providers in LMICs with a focus on primary data collection as opposed to further literature reviews.

A greater focus on interventions beyond cybersecurity training must be investigated. Expanding primary research methodologies beyond staff surveys and questionnaires also offers the opportunity to capture more information on technical interventions related to cybersecurity. Additionally, further research should evaluate the culture change around the importance of cybersecurity and implications for impact and outcomes. We believe greater research is required, using learnings from the field of implementation science and seeking to build a robust and generalizable evidence base to inform implementation practice [54].

This review also identified the important role of national and regional policy and legislation as an enabler of cybersecurity intervention implementation. Clear national legislation and regulation, with accompanying guidance on cybersecurity, provides health care organizations with a mandate and direction to develop and implement organizational policies and interventions accordingly. It is also recommended that health care organizations in LMICs collaborate with government stakeholders in the implementation of cybersecurity interventions where possible, to enable government stakeholders to understand the practice-level challenges toward improved policy and guidance long term.

### Conclusion

This scoping review presents a comprehensive description of cybersecurity interventions implemented in LMICs. The small number of studies identified highlights the limited body of research published in this topic area and shows large heterogeneity in interventions, research objectives, methods, and outcome measures used. Consistent with wider literature on the impacts of cybersecurity interventions, the impact of cybersecurity interventions on the likelihood and impact of cyberattacks in health care organizations remains unclear, making a reliable analysis of evidence difficult. Nonetheless, it is important to continue research to explore the impact, although complex and difficult to assess, to enable targeted cybersecurity initiatives to be used for the benefit of patients, providers, and health systems.

Current evidence points to clear national legislation and policies, supported in organizations by active monitoring, evaluation, and learning, and provider-level staff engagement and training as facilitators of cybersecurity interventions. Staff perception of the importance of cybersecurity, intervention ease of use, and security and reliability of interventions facilitate successful implementation. However, a lack of policy and guidance for organizations, a lack of knowledge among staff and health ICT experts, and the costs of implementation challenge greater implementation of cybersecurity initiatives in health care organizations. Future research should directly evaluate cybersecurity interventions and expand methodologies to build a robust and generalizable evidence base to inform policy and practice.

## Appendix

Multimedia Appendix 1 Search terms.

Multimedia Appendix 2 PRISMA-ScR checklist.

## Abbreviations

COBIT: Control Objectives for Information and Related Technologies
ECHO: Essentials of Cybersecurity in Health Care Organizations
HIC: high-income country
ICT: information and communication technology
LMIC: low- and middle-income country
NHS: National Health Service
NIST: National Institute of Standards and Technology
PRISMA: Preferred Reporting Items for Systematic Reviews and Meta-Analyses
WHO: World Health Organization

## Data Availability

The data sets generated during and/or analyzed during this study are available from the corresponding author on reasonable request.

## References

[1] Gopal G, Suter-Crazzolara C, Toldo L, Eberhardt W (2019). Digital transformation in healthcare - architectures of present and future information technologies. Clin Chem Lab Med.

[2] Coventry L, Branley D (2018). Cybersecurity in healthcare: A narrative review of trends, threats and ways forward. Maturitas.

[3] Cyber Threats 2020: A Year in Retrospect. PWC.

[4] Attack. National Institute of Standards and Technology (NIST) Information Technology Laboratory Computer Security Resource Center.

[5] Martin G, Martin P, Hankin C, Darzi A, Kinross J (2017). Cybersecurity and healthcare: how safe are we?. BMJ.

[6] Neprash HT, McGlave CC, Cross DA, Virnig BA, Puskarich MA, Huling JD, Rozenshtein AZ, Nikpay SS (2022). Trends in ransomware attacks on US hospitals, clinics, and other health care delivery organizations, 2016-2021. JAMA Health Forum.

[7] Javaid M, Haleem A, Singh RP, Suman R (2023). Towards insighting cybersecurity for healthcare domains: A comprehensive review of recent practices and trends. Cyber Security and Applications.

[8] (2018). Investigation: WannaCry cyber attack and the NHS. National Audit Office.

[9] Corera G (2021). Irish health cyber-attack could have been even worse, report says. BBC.

[10] Devi S (2023). Cyber-attacks on health-care systems. The Lancet Oncology.

[11] O’Brien N, Ghafur S, Durkin M (2021). Cybersecurity in health is an urgent patient safety concern: We can learn from existing patient safety improvement strategies to address it. Journal of Patient Safety and Risk Management.

[12] Ghafur S, Kristensen S, Honeyford K, Martin G, Darzi A, Aylin P (2019). A retrospective impact analysis of the WannaCry cyberattack on the NHS. NPJ Digit Med.

[13] Jones S, Moulton C, Swift S, Molyneux P, Black S, Mason N, Oakley R, Mann C (2022). Association between delays to patient admission from the emergency department and all-cause 30-day mortality. Emerg Med J.

[14] Herrod PJJ, Adiamah A, Boyd-Carson H, Daliya P, El-Sharkawy AM, Sarmah PB, Hossain T, Couch J, Sian TS, Wragg A, Andrew DR, Parsons SL, Lobo DN, WES-Pi Study Group on behalf of the East Midlands Surgical Academic Network (EMSAN), WES-Pi Study Group (2019). Winter cancellations of elective surgical procedures in the UK: a questionnaire survey of patients on the economic and psychological impact. BMJ Open.

[15] Holeman I, Cookson TP, Pagliari C (2016). Digital technology for health sector governance in low and middle income countries: a scoping review. J Glob Health.

[16] Leesa-Nguansuk S (2021). Warning over cyberattack threats. Bangkok Post.

[17] (2020). South Africa's Life Healthcare hit by cyber attack. Reuters.

[18] Moher D, Liberati A, Tetzlaff J, Altman DG, the PRISMA Group (2009). Reprint—Preferred Reporting Items for Systematic Reviews and Meta-Analyses: The PRISMA Statement. Physical Therapy.

[19] World Bank Country and Lending Groups. The World Bank.

[20] Lencucha R, Neupane S (2022). The use, misuse and overuse of the 'low-income and middle-income countries' category. BMJ Glob Health.

[21] O'Brien N, Grass E, Martin G, Durkin M, Darzi A, Ghafur S (2020). Developing a globally applicable cybersecurity framework for healthcare: a Delphi consensus study. BMJ Innov.

[22] O'Brien N, Martin G, Grass E, Durkin M, Ghafur S (2020). Safeguarding our Healthcare Systems: A Global Framework for Cybersecurity. World Innovation Summit for Health.

[23] Implementation Know-how Briefs to Support Countries to Prioritize, Connect and Scale for a Digital-in-Health Future. The World Bank.

[24] Ali Alferjanya MAO, Musaed Al-mwald MN, Alias RB (2022). The effect of cyber security knowledge on employees' personal growth: An empirical study in private hospitals in libya and yemen. Health Education and Health Promotion.

[25] Ayatollahi H, Shagerdi G (2017). Information security risk assessment in hospitals. Open Med Inform J.

[26] Chuma K, Ngoepe M (2021). Security of electronic personal health information in a public hospital in South Africa. Information Security Journal: A Global Perspective.

[27] Daraghmeh R, Brown R (2021). A big data maturity model for electronic health records in hospitals.

[28] Dönmez E, Kitapçı NŞ, Kitapçı OC, Yay M, Aksu PK, Köksal L, Mumcu G (2020). Readiness for health information technology is associated to information security in healthcare institutions. Acta Inform Med.

[29] Hou Y, Gao P, Nicholson B (2018). Understanding organisational responses to regulative pressures in information security management: The case of a Chinese hospital. Technological Forecasting and Social Change.

[30] Jara HLS, Navarro HBP, Armas-Aguirre J, Iano Y, Saotome O, Kemper G, Mendes de Seixas AC, Gomes de Oliveira G (2021). Cybersecurity and Privacy Capabilities Model for Data Management Against Cyber-Attacks in the Health Sector. Proceedings of the 6th Brazilian Technology Symposium (BTSym’20). BTSym 2020. Smart Innovation, Systems and Technologies, vol 233.

[31] Kandabongee Yeng P, Yang BM, Stolt Pedersen M (2023). Assessing cyber-security compliance level in paperless hospitals: An ethnographic approach. https://ieeexplore.ieee.org/document/10061936.

[32] Khac Hai N, Lawpoolsri S, Jittamala P, Thi Thu Huong P, Kaewkungwal J (2017). Practices in security and confidentiality of HIV/AIDS patients' information: A national survey among staff at HIV outpatient clinics in Vietnam. PLoS One.

[33] Maeko ME, Van Der Haar DT (2018). A framework for user awareness and acceptance of smart card and fingerprint-based access control to medical information systems in South Africa. https://ieeexplore.ieee.org/document/8417340.

[34] Meng W, Choo K, Furnell S, Vasilakos A, Probst C (2018). Towards bayesian-based trust management for insider attacks in healthcare software-defined networks. T-NSM.

[35] Nistrina K, Bin Bon HAT (2019). Information security for hospital information system using COBIT 5 framework. http://ieomsociety.org/ieom2019/papers/767.pdf.

[36] Quimiz-Moreira M, Zambrano-Romero W, Moreira-Zambrano C, Mendoza-Zambrano M, Cedeño-Palma E, Botto-Tobar MS, Gómez O, Rosero Miranda R, Díaz Cadena A, Montes León S, Luna-Encalada W (2022). Cybersecurity mechanisms for information security in patients of public hospitals in Ecuador. Trends in Artificial Intelligence and Computer Engineering. ICAETT 2021. Lecture Notes in Networks and Systems, vol 407.

[37] Rijo R, Martinho R, Aparecida Oliveira A, Alves D, Silveira Nogueira Reis Z, Santos-Pereira C, Correia ME, Antunes LF, Cruz-Correia RJ (2020). Profiling IT security and interoperability in brazilian health organisations from a business perspective. International Journal of E-Health and Medical Communications (IJEHMC).

[38] Serrano Rojas AJ, Paniura Valencia EF, Armas-Aguirre J, Madrid Molina JM (2022). Cybersecurity maturity model for the protection and privacy of personal health data.

[39] Sanchez Rubio CJ, Villacorta GG, Choque JO, Armas-Aguirre J (2022). Personal health data: A security capabilities model to prevent data leakage in big data environments.

[40] Sarbaz M, Manouchehri Monazah F, Banaye Yazdipour A, Kimiafar K (2019). Views of health information management staff on non-technical security management factors, Mashhad, Iran. Stud Health Technol Inform.

[41] Kencana Sari P, Wuri Handayani P, Nizar Hidayanto A, Wiranda Busro P (2023). How information security management systems influence the healthcare professionals’ security behavior in a public hospital in Indonesia. IJIKM.

[42] Singh I, Singh Y (2022). Cyber-security knowledge and practice of nurses in private hospitals in northern Durban, Kwazulu-Natal. Journal of Theoretical and Applied Information Technology.

[43] Upadhyaya P, Upadhyay SK, Subedi B, Subedi B, Gaire A (2019). Revolutionizing healthcare systems of a developing country using blockchain. https://ieeexplore.ieee.org/document/8782417.

[44] Alhuwail D, Al-Jafar E, Abdulsalam Y, AlDuaij S (2021). Information security awareness and behaviors of health care professionals at public health care facilities. Appl Clin Inform.

[45] Nifakos S, Chandramouli K, Nikolaou C, Papachristou P, Koch S, Panaousis E, Bonacina S (2021). Influence of human factors on cyber security within healthcare organisations: a systematic review. Sensors (Basel).

[46] Arain MA, Tarraf R, Ahmad A (2019). Assessing staff awareness and effectiveness of educational training on IT security and privacy in a large healthcare organization. J Multidiscip Healthc.

[47] (2021). What Good Looks Like framework. NHS England.

[48] He Y, Aliyu A, Evans M, Luo C (2021). Health care cybersecurity challenges and solutions under the climate of COVID-19: scoping review. J Med Internet Res.

[49] Feeley A, Lee M, Crowley M, Feeley I, Roopnarinesingh R, Geraghty S, Cosgrave B, Sheehan E, Merghani K (2022). Under viral attack: An orthopaedic response to challenges faced by regional referral centres during a national cyber-attack. Surgeon.

[50] Coventry L, Branley-Bell D, Sillence E, Magalini S, Mari P, Magkanaraki A, Anastasopoulou K (2020). Cyber-Risk in Healthcare: Exploring Facilitators and Barriers to Secure Behaviour.

[51] Branley-Bell D, Coventry L, Sillence E (2021). Promoting cybersecurity culture change in healthcare. Northumbria University Research Portal.

[52] Wilner A, Luce H, Ouellet E, Williams O, Costa N (2022). From public health to cyber hygiene: Cybersecurity and Canada’s healthcare sector. International Journal.

[53] Pham M, Rajić A, Greig J, Sargeant J, Papadopoulos A, McEwen S (2014). A scoping review of scoping reviews: advancing the approach and enhancing the consistency. Res Synth Methods.

[54] Klaic M, Kapp S, Hudson P, Chapman W, Denehy L, Story D, Francis JJ (2022). Implementability of healthcare interventions: an overview of reviews and development of a conceptual framework. Implement Sci.

